# MiR-141-3p ameliorates RIPK1-mediated necroptosis of intestinal epithelial cells in necrotizing enterocolitis

**DOI:** 10.18632/aging.103608

**Published:** 2020-07-23

**Authors:** Xiang Li, Ying Wang, Yijiang Wang, Xingbo He

**Affiliations:** 1 Department of Pediatrics, The Second Children and Women’s Healthcare of Jinan City, Jinan, Shandong Province, China; 2 Department of Emergency, Jinan Children’s Hospital, Jinan, Shandong Province, China

**Keywords:** necrotizing enterocolitis, MicroRNA-141-3p, receptor interacting protein kinase 1, necroptosis, lipopolysaccharide

## Abstract

Aim: To explore the effects of miR-141-3p on intestinal epithelial cells in necrotizing enterocolitis and the underlying mechanism.

Results: The expression of miR-141-3p was significantly downregulated in serum samples of patients with NEC and LPS-treated Caco-2 cells. The *in vitro* assays showed that miR-141-3p mimics inhibited expression of IL-6 and TNF-α and reduced PI positive rate of the LPS-treated Caco-2 cells. Next, receptor interacting protein kinase 1 (RIPK1) was identified as the downstream molecule of miR-141-3p, and RIPK1 overexpression aggravated LPS-induced Caco-2 cell injury, which was ameliorated by miR-141-3p mimics. Finally, we found miR-141-3p mimics inhibited upregulation of necroptosis-related molecules and interaction of RIPK1 and RIPK3 in LPS-treated Caco-2 cells.

Conclusion: Our research indicated that miR-141-3p protected intestinal epithelial cells from LPS damage by suppressing RIPK1-mediated inflammation and necroptosis, providing an alternative perspective to explore the pathogenesis of NEC.

Methods: Quantitative real time-polymerase chain reaction (qRT-PCR) was used to detect the expression of miR-141-3p in serum samples of participants and lipopolysaccharide (LPS)-treated Caco-2 cells. Cell Counting Kit-8 (CCK-8) assay, Propidium Iodide (PI) staining and detection of inflammatory cytokines were performed to evaluate the role of miR-141-3p in LPS-treated Caco-2 cells. TargetScanHuman database and luciferase reporter gene assay were utilized to confirm the direct downstream molecule of miR-141-3p. Western blot analysis was used to explore the mechanism.

## INTRODUCTION

Necrotizing enterocolitis (NEC) is one of common diseases that causes death of preemies and neonates in neonatal intensive care unit [1]. It is noteworthy that the mortality of patients with NEC is about 20%-30% [2]. Although the exact mechanism of NEC is still unclear, accumulating evidence has reported that bacterial infection of intestine is one of the predominant factors contributing to pathogenesis of NEC [3, 4]. Generally, abnormal bacteria invade intestinal epithelial cells to cause cell death and inflammation, therefore aggravating intestinal and systemic injury. Thus, suppressing cell death and inflammation is a potential strategy for treatment of NEC.

Clinical data has demonstrated that intestinal dysbiosis and colonization of various bacteria in intestine contribute to occurrence and development of NEC [5, 6]. Lipopolysaccharide (LPS), a component of Gram-negative bacteria, is an inducer of NEC [7]. It has been reported that an *in vitro* NEC model could be established by treating intestinal epithelial cells with LPS [8, 9]. A recent study showed that milk exosomes could inhibit inflammation and apoptosis of LPS-treated intestinal epithelial cells by suppressing nuclear factor kappa-B (NF-kB) and p53, respectively [10]. In addition, intraperitoneal injection with LPS in prenatal mice promoted the expression of tumor necrosis factor (TNF) and decreased the microvascular density in intestines of the neonatal mice, which facilitated the severity of NEC [11]. Therefore, LPS might aggravate intestinal injury through multiple aspects.

MicroRNAs (MiRNAs) are a cluster of small molecules that exist abundantly in eukaryotic organisms [12]. Plenty of evidence has demonstrated that miRNAs could regulate biological functions of cells by targeting specific mRNAs, and targeting miRNAs is a potential therapeutic approach for treatment of multiple diseases [13, 14]. Recent studies have reported the aberrant expression of miRNAs exists in NEC. Xu et al [15] found that a total of 118 miRNAs were expressed differentially in rats with NEC, which potentially targeted some functional molecules to modulate progression of NEC. Clinical samples from infants with NEC showed upregulation of miR-1290 in plasma, which could be regarded as a potential biomarker of NEC [16]. An *in vitro* study revealed that miR-431 was significantly overexpressed in infants with NEC and LPS-treated Caco-2 cells, and promoted apoptosis and inflammation of Caco-2 cells [9]. A recent study indicated the downregulation of miR-141-3p in newborns with NEC [17]. However, the role of miR-141-3p in pathogenesis of NEC is unknown. It has been reported that miR-141-3p facilitates cell survival. Zhu et al [18] found that miR-141-3p protected nasal epithelial cells from LPS damage. In addition, miR-141-3p alleviates inflammatory response to protect cardiomyocytes [19] and microglial cells [20]. Therefore, we speculated the potential role of miR-141-3p in LPS-treated intestinal epithelial cells might contribute to the pathogenesis of NEC.

Herein, our research revealed downregulation of miR-141-3p in NEC, and receptor-interacting protein kinase 1 (RIPK1) functioned as the direct downstream molecule of miR-141-3p to aggravate intestinal epithelial cell injury by activating receptor-interacting protein kinase 3 (RIPK3)/mixed lineage kinase domain like pseudokinase (MLKL)-mediated necroptotic signaling pathway, which might contribute to the pathogenesis of NEC.

## RESULTS

### MiR-141-3p was downregulated in NEC

**Figure 1:**
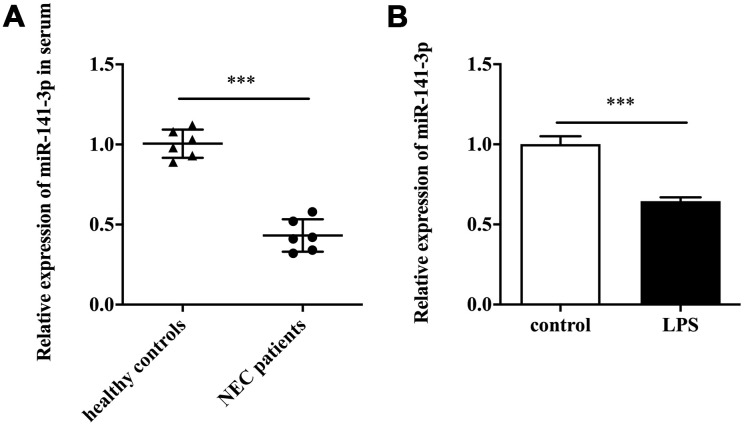
**MiR-141-3p was downregulated in NEC.** (**A**) The expression of miR-141-3p in serum samples of newborns with NEC. (**B**) The expression of miR-141-3p in NEC cell model. ^***^p<0.001.

Firstly, we detected miR-141-3p expression in clinical samples. The qRT-PCR result showed that serum samples of patients with NEC existed a lower expression of miR-141-3p compared with healthy controls (Figure 1A). Next, we established the NEC cell model by treating Caco-2 cells with LPS. The *in vitro* assay showed that LPS induced downregulation of miR-141-3p (Figure 1B).

### MiR-141-3p downregulation participated in LPS-induced Caco-2 cell injury

**Figure 2:**
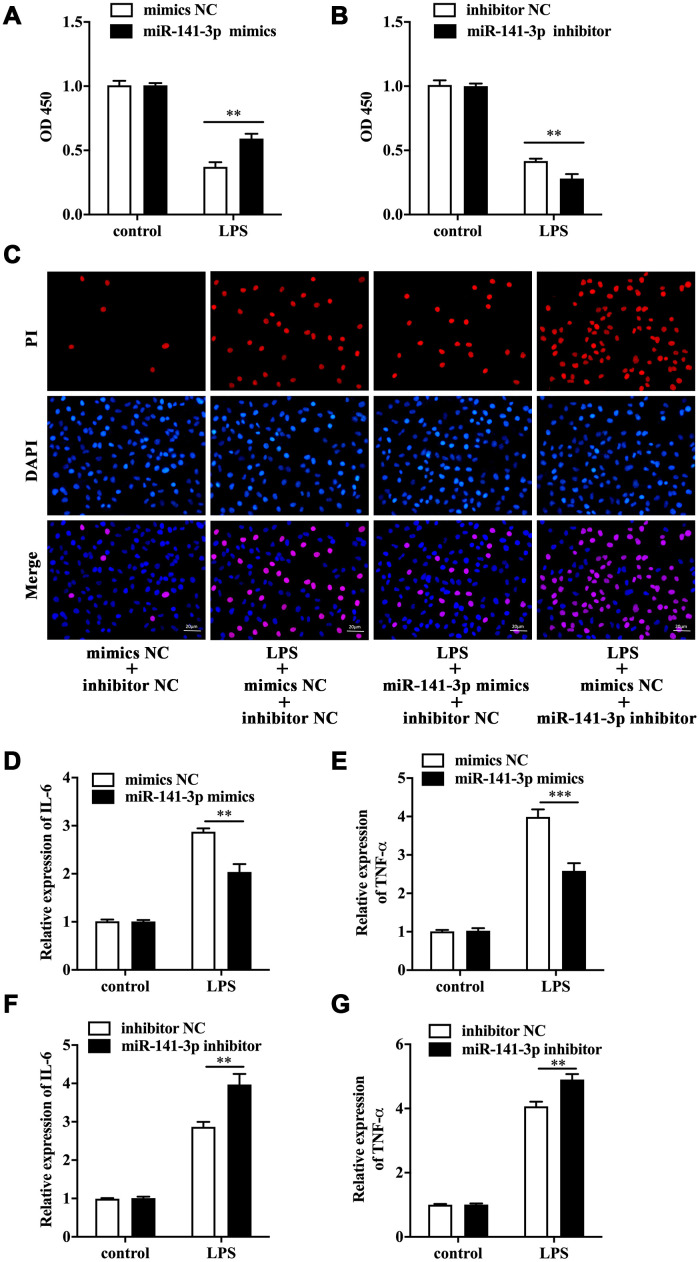
**MiR-141-3p downregulation participated in LPS-induced Caco-2 cell injury.** Cell viability of LPS-treated Caco-2 cells in the presence of miR-141-3p mimics (**A**) or miR-141-3p inhibitor (**B**). (**C**) PI positive staining of LPS-treated Caco-2 cells with miR-141-3p mimics transfection or miR-141-3p inhibitor transfection. The mRNA level of IL-6 (**D**) and TNF-α (**E**) in LPS-treated Caco-2 cells with miR-141-3p mimics transfection. The expression of IL-6 (**F**) and TNF-α (**G**) in LPS-treated Caco-2 cells with miR-141-3p inhibitor transfection. Scale bar = 20 μm. ^**^p<0.01, ^***^p<0.001.

To explore the biological role of miR-141-3p in intestinal epithelial cells of NEC, we conducted cell transfection in Caco-2 cells with miR-141-3p mimics and miR-141-3p inhibitor in the presence or absence of LPS. As was shown in Figure 2A, LPS reduced cell viability of Caco-2 cells by measuring OD 450, which was partially reversed by miR-141-3p mimics. Conversely, miR-141-3p inhibitor could further reduce the decreased cell viability of LPS-treated Caco-2 cells (Figure 2B). The fluorescence analysis showed that LPS increased PI positive rate of Caco-2 cells, which was suppressed by miR-141-3p mimics (Figure 2C). In addition, miR-141-3p inhibitor could further increase LPS-induced PI positive cells (Figure 2C). The qRT-PCR analysis revealed that LPS-induced upregulation of IL-6 and TNF-α was restrained by miR-141-3p mimics (Figure 2D and 2E). However, miR-141-3p inhibitor further facilitated upregulation of IL-6 and TNF-α in LPS-treated Caco-2 cells (Figure 2F and 2G). The above results suggested that miR-141-3p reduced necrosis and inflammation in LPS-treated Caco-2 cells.

### RIPK1 functioned downstream of miR-141-3p and aggravated LPS-induced Caco-2 cell injury

**Figure 3:**
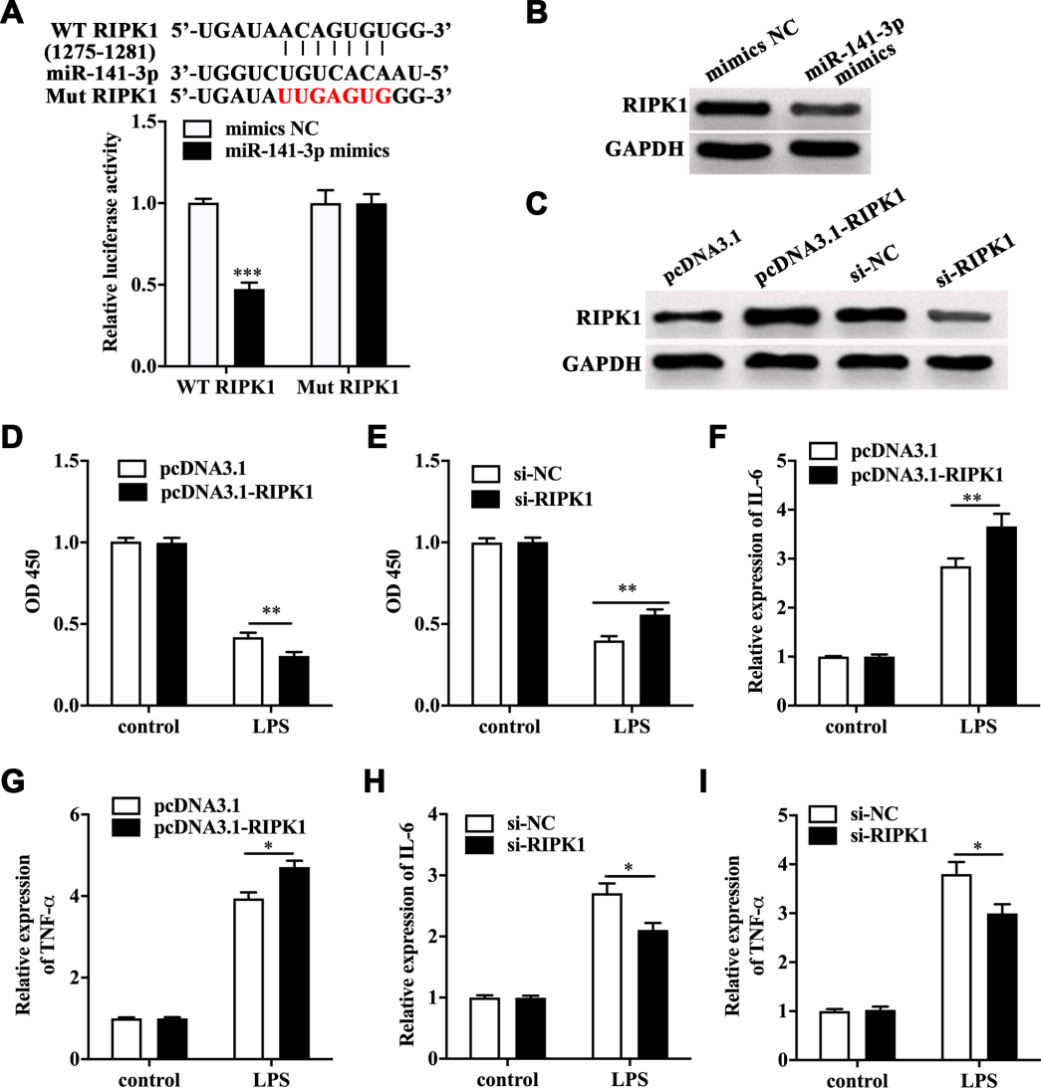
**RIPK1 was the direct target of miR-141-3p and aggravated LPS-induced Caco-2 cell injury.** (**A**) The predicted binding sites between miR-141-3p and RIPK1, and Luciferase reporter gene assay was performed to test the interaction between miR-141-3p and RIPK1. (**B**) The protein level of RIPK1 in miR-141-3p mimics-transfected Caco-2 cells. (**C**) The expression of RIPK1 in Caco-2 cells with RIPK1 overexpression and knockdown. Cell viability of LPS-treated Caco-2 cells with RIPK1 overexpression (**D**) or knockdown (**E**). The expression of IL-6 (**F**) and TNF-α (**G**) in LPS-treated Caco-2 cells with RIPK1 overexpression. The mRNA level of IL-6 (**H**) and TNF-α (**I**) in LPS-treated Caco-2 cells with RIPK1 siRNA transfection. ^*^p<0.05, ^**^p<0.01, ^***^p<0.001.

Next, TargetScanHuman 7.2 database was utilized to screen the target of miR-141-3p. We found RIPK1, a key mediator of necroptosis, was one of the potential targets of miR-141-3p. The luciferase reporter gene assay showed a remarkably lower luciferase activity in Caco-2 cells co-transfected with wild type RIPK1 and miR-141-3p mimics (Figure 3A). Notably, miR-141-3p mimics inhibited the expression of RIPK1 in Caco-2 cells (Figure 3B). RIPK1 overexpression and knockdown were performed in Caco-2 cells by transfecting with pcDNA3.1-RIPK1 and RIPK1 siRNA, and the efficacy was measured by Western blot (Figure 3C). CCK-8 assay indicated that RIPK1 overexpression aggravated the decreased cell viability in LPS-treated Caco-2 cells; however, RIPK1 knockdown reversed the decreased cell viability in LPS-treated Caco-2 cells (Figure 3D and 3E). Moreover, we found that RIPK1 overexpression could increase the upregulation of IL-6 and TNF-α (Figure 3F and 3G); while RIPK1 siRNA suppressed the increased expression of IL-6 and TNF-α in LPS-treated Caco-2 cells (Figure 3H and 3I).

### LPS induced RIPK1-mediated necroptosis of Caco-2 cells

**Figure 4:**
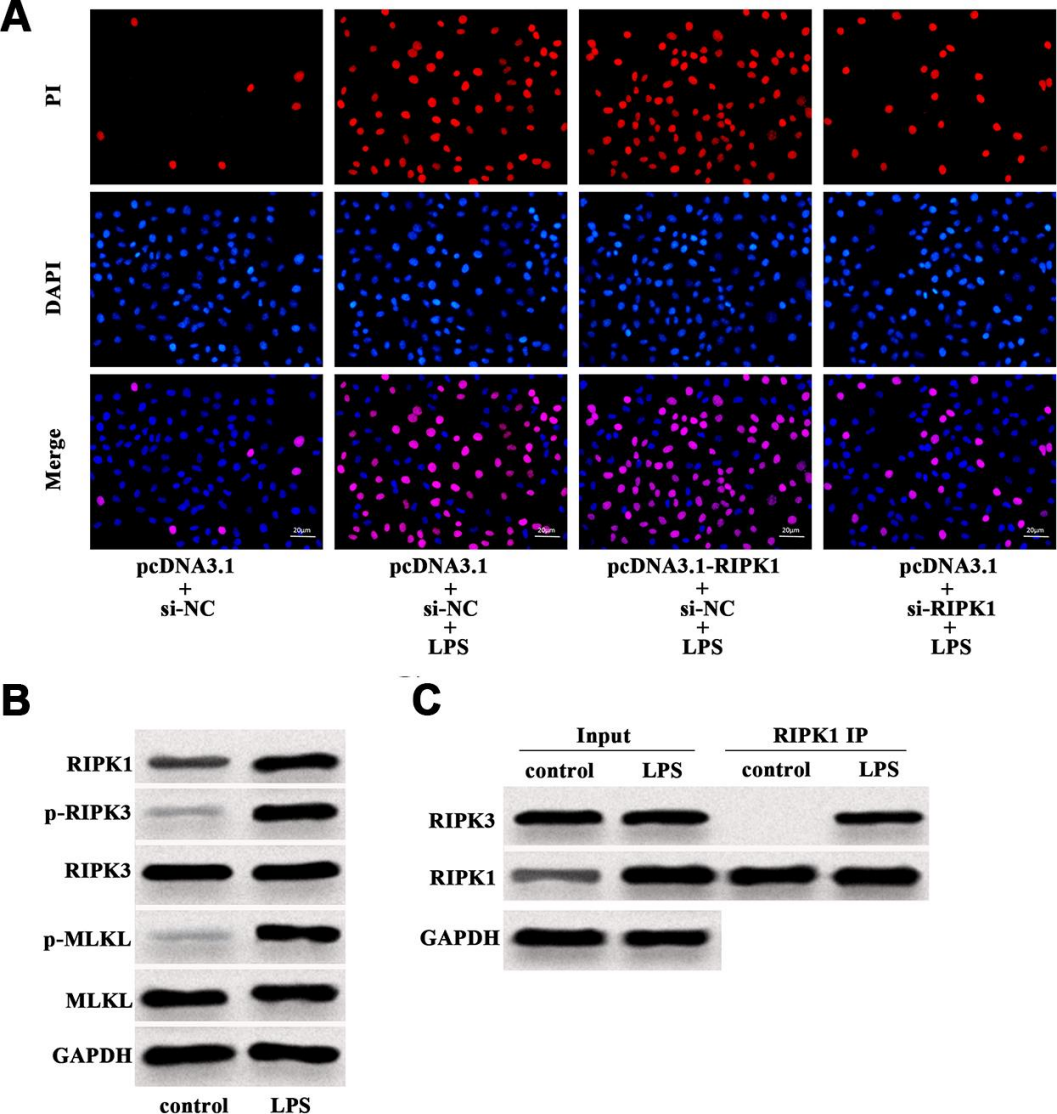
**LPS induced RIPK1-mediated necroptosis of Caco-2 cells.** (**A**) PI positive staining of LPS-treated Caco-2 cells with pcDNA3.1-RIPK1 transfection or RIPK1 siRNA transfection. (**B**) The protein level of RIPK1, p-RIPK3, RIPK3, p-MLKL and MLKL in LPS-treated Caco-2 cells. (**C**) IP assay was conducted to determine the interaction between RIPK1 and RIPK3 in LPS-treated Caco-2 cells. Scale bar = 20 μm.

Further investigation revealed that LPS-induced increase of PI positive cells was augmented by RIPK1 overexpression, but suppressed by RIPK1 knockdown (Figure 4A). Western blot analysis showed that LPS facilitated the expression of RIPK1, phosphorylated RIPK3 (p-RIPK3) and phosphorylated MLKL (p-MLKL) (Figure 4B), indicating the activation of necroptotic signaling pathway. In addition, LPS treatment had no influence on the expression of RIPK3 and MLKL in Caco-2 cells (Figure 4B). We also performed IP assay and found that LPS treatment could promote the interaction of RIPK1 and RIPK3 in Caco-2 cells (Figure 4C). These results suggested that RIPK1-mediated necroptosis contributed to LPS-induced intestinal epithelial cell death.

### MiR-141-3p inhibited RIPK1-mediated necroptosis to reduce LPS-induced Caco-2 cell injury

**Figure 5:**
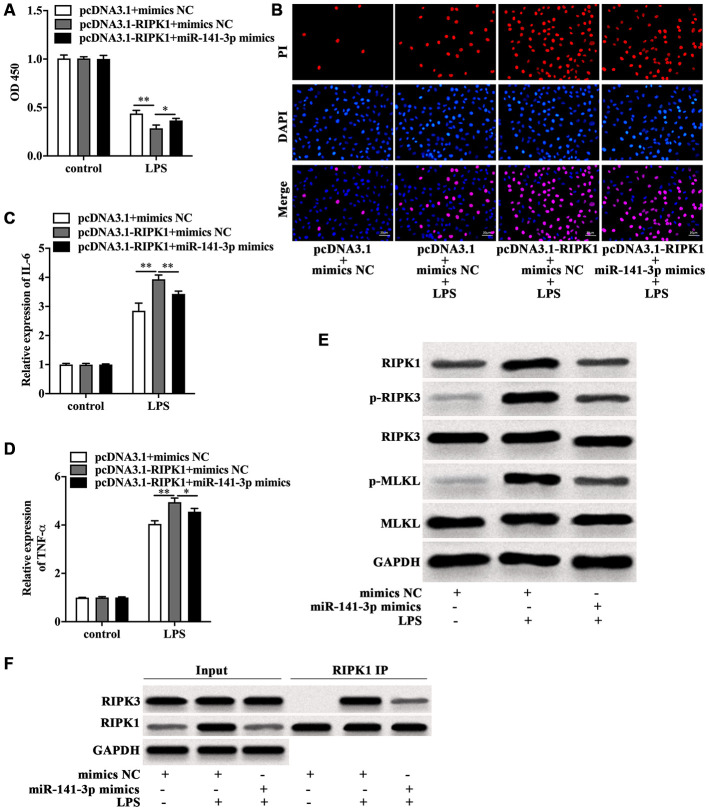
**MiR-141-3p inhibited RIPK1-mediated necroptosis to reduce LPS-induced Caco-2 cell injury.** Cell viability (**A**), PI positive staining (**B**), IL-6 mRNA level (**C**), TNF-α mRNA level (**D**) of LPS-treated Caco-2 cells with RIPK1 overexpression in the presence of miR-141-3p mimics. (**E**) The protein level of RIPK1, p-RIPK3, RIPK3, p-MLKL and MLKL in LPS-treated Caco-2 cells in the presence or absence of miR-141-3p mimics. (**F**) IP assay was carried out to detect the interaction between RIPK1 and RIPK3 in LPS-treated Caco-2 cells in the presence or absence of miR-141-3p mimics. Scale bar = 20 μm. ^*^p<0.05, ^**^p<0.01.

Next, the role of miR-141-3p in RIPK1-mediated Caco-2 cell injury was explored. CCK-8 assay indicated that RIPK1 overexpression magnified the decreased cell viability of LPS-treated Caco-2 cells, which was rescued by miR-141-3p mimics (Figure 5A). In parallel, miR-141-3p mimics reduced PI positive rate of LPS-treated Caco-2 cells in the presence of RIPK1 overexpression (Figure 5B). Additionally, miR-141-3p mimics inhibited LPS-induced elevated expression of IL-6 and TNF-α in pcDNA3.1-RIPK1-transfected Caco-2 cells (Figure 5C and 5D). Western blot analysis revealed that miR-141-3p mimics reversed the upregulation of RIPK1, p-RIPK3 and p-MLKL induced by LPS (Figure 5E). Moreover, miR-141-3p mimics could reduce the interaction of RIPK1 and RIPK3 in LPS-treated Caco-2 cells (Figure 5F), indicating miR-141-3p ameliorated intestinal epithelial cell injury by targeting LPS-induced RIPK1-mediated necroptosis.

## DISCUSSION

Our research indicated the downregulation of miR-141-3p in serum of patients with NEC and in the *in vitro* NEC model. The *in vitro* assays showed that the decreased miR-141-3p aggravated cell death and inflammation of LPS-treated intestinal epithelial cells. Further investigation revealed that RIPK1 was the target of miR-141-3p, and miR-141-3p alleviated LPS-induced intestinal epithelial cell injury by inhibiting RIPK1-mediated necroptosis and inflammation.

As a group of non-coding RNAs, miRNAs could participate in the occurrence and progression of multiple diseases by regulating functional proteins, which could be potential treatment targets [13, 14]. Increasing evidence has demonstrated that aberrant expression of miRNAs plays a crucial role in pathogenesis of inflammation-related intestinal diseases [21]. A *in vivo* study revealed that miR-21 knockout regulated the homeostasis of intestinal microbiota to reduce intestinal damage in mice with dextran sodium sulphate-induced colitis [22]. In inflammatory bowel diseases, miR-31 deletion deteriorated intestinal injury by inhibiting intestinal epithelial cell regeneration and promoting inflammatory response in mice [23]. Cai et al [24] found that downregulation of miR-141 in ulcerative colitis promoted the expression of CXCL5 to aggravate inflammatory response of intestinal epithelial cells. However, only a few studies concentrate on the effects of miRNAs on the pathogenesis of NEC. A recent research identified upregulation of miR-1290 in plasma as a potentially diagnostic biomarker of NEC [16]. Additionally, Wu et al [9] reported that miR-431 aggravated inflammatory damage of LPS-treated intestinal epithelial cells by targeting FOXA1, based on the previous research that suggested a correlation between miR-431 and FOXA1 in a potential mechanism of pathogenesis of NEC [25]. Herein, our study indicated the decreased expression of miR-141-3p in NEC, which coincided with the previous findings [17]. However, whether miR-141-3p contributes to pathogenesis of NEC needs to be elucidated.

Accumulating evidence suggests that miR-141-3p and its precursor miR-141 exert protective effects on cell damage. A previous study showed that the decreased expression of miR-141-3p was detected in rats with autoimmune myocarditis, and miR-141-3p overexpression lessened the expression of inflammatory cytokines and infiltration of inflammatory cells by inhibiting STAT4 to improve cardiac function of the experimental rats [19]. In LPS-treated microglial cells, miR-141-3p overexpression directly inhibited HMGB1 to relieve inflammation [20]. In addition, miR-141 could target different molecules to reduce cell death and ameliorate inflammatory response in Crohn’s disease [26], lung inflammation [27] and prostatitis [28]. In our research, RIPK1 was identified as the direct target of miR-141-3p. Moreover, miR-141-3p protected Caco-2 cells from LPS damage by suppressing RIPK1-mediated necroptosis and expression of inflammatory cytokines.

Necroptosis, a novel programmed cell death, is a crucial cellular behavior that plays an indispensable role in embryogenesis, organogenesis, and occurrence and development of diseases [29, 30]. Although Necroptosis shares familiarity of morphological changes with necrosis, it could be regulated by RIPK1 [31, 32], which is a significantly different feature from necrosis. Generally, TNF-α is a classical inducer of necroptosis that binds to its receptor to recruit RIPK1, which interacts with RIPK3 to form necrosome and phosphorylate MLKL to mediate necroptosis in the absence of caspases-8 [31, 33, 34]. Therefore, targeting RIPK1, RIPK3 and MLKL could dramatically block necroptosis [32, 34]. The *in vitro* [35] and *in vivo* [36] assays manifested that LPS could induce necroptosis of intestinal epithelial cells. A recent research indicated the existence of necroptosis in clinical samples and mouse models of NEC [37]. However, the exact mechanism is unclear. We found upregulation of RIPK1 activated the downstream molecules and promoted necrosome formation in LPS-treated Caco-2 cells, and miR-141-3p inhibited RIPK1 to reverse intestinal epithelial cell damage, which provided an upstream mechanism involving regulation of RIPK1-mediated necroptosis in NEC. Taken together, our study uncovered miR-141-3p functioned as a protective molecule in NEC-related intestinal epithelial cell injury that involved RIPK1-mediated necroptosis and inflammation, providing an alternative perspective to further develop potential strategies for treatment of NEC.

However, we only detected the expression of miR-141-3p in infection-mediated NEC in clinical samples and LPS-treated Caco-2 cells. Whether the decreased expression of miR-141-3p existed in NEC caused by the other factors, such as premature birth, low birth weight and improper feeding, deserved further investigation. Moreover, we only used Caco-2 cell line to conduct the experiment, establishing an *in vitro* NEC model by utilizing primary intestinal epithelial cells and an animal NEC model might be more valuable.

## MATERIALS AND METHODS

### Isolation of serum samples

The serum samples were acquired from the newborns with NEC (n=6) and healthy controls (n=6). The diagnosis of NEC was based on the criterial of Bell et al [38]. The pregnant women, aged between 23-30 years old, were primipara without underlying diseases, and gave birth during 37-41 weeks’ gestation. The newborns were born without birth defects and other diseases, and breastfed. All the procedures were authorized by the Second Children and Women’s Healthcare of Jinan City. Before collecting the samples, the investigators had informed guardians of the participants of the purpose and methods of this research, and all the guardians signed the informed consent. The fresh blood was centrifuged at a speed of 3000 rpm for 12 min to drain the supernatant carefully. This study was reviewed and approved by the Ethics Committee of the Second Children and Women’s Healthcare of Jinan City.

### Cell culture and treatment

Human Caco-2 cells were obtained from ATCC and cultured in DMEM (ThermoFisherScientific, USA) containing 10% fetal bovine serum (Gibco, USA) and 10 mM HEPES (ThermoFisherScientific, USA). To establish the *in vitro* NEC model, we treated Caco-2 cells with 100 μg/ml LPS (Sigma-Aldrich, USA) in this study.

### Quantitative real time PCR (qRT-PCR)

The total RNA was obtained by using TRIzol (Ambion, USA). SuperScript III (Invitrogen, USA) was used to obtain cDNA. SYBR Premix Ex Taq II (Takara, Japan) was utilized to perform qRT-PCR. The primer sequences were shown in Table 1. U6 and GAPDH were used as the internal control. All of the primers were synthesized and purchased from Sangon (China).

**Table 1:** Sequences of primers, siRNAs, microRNA mimic, and microRNA inhibitor used in this study.

**Gene**	**Sequence (5’-3’)**
MiR-141-3p	Forward: GGTCCTAACACTGTCTGGTAAAGTGG
	Reverse: CCAGTGCAGGGTCCGAGGT
U6	Forward: TGCGGGTGCTCGCTTCGGCAGC
	Reverse: CCAGTGCAGGGTCCGAGGT
GAPDH	Forward: AGACCACAGTCCATGCCATC
	Reverse: CAGGGCCCTTTTTCTGAGCC
TNF-α	Forward: GACAGCAGAGGACCAGCTAA
	Reverse: AGCTGTCATATTTCCCGCTCT
IL-6	Forward: CATCCCATAGCCCAGAGCAT
	Reverse: CAGGCTGGCATTTGTGGTTG
RIPK1 siRNA	Forward: GAAUGAGGCUUACAACAGTT
	Reverse: CUGUUGUAAGCCUCAUUCTT
Si-NC	AAUUCUCCGAACGUGUCACGU
MiR-141-3p mimic	Forward: UAUUGCACAUUACUAAGUUGCA
	Reverse: CAACUUAGUAAUGUGCAAUAUU
Mimic NC	UUCUCCGAACGUGUCACUGUU
MiR-141-3p inhibitor	UGCAACUUAGUAAUGUGCAAUA
Inhibitor NC	CAGUACUUUUGUGUAGUACAA

U6: U6 small nuclear RNA; GAPDH: Glyceraldehyde-3-phosphate dehydrogenase; TNF-α: Tumor necrosis factor alpha; IL-6: Interleukin 6; siRNA: Small interfering RNA; NC: Negative control.

### Cell transfection

RIPK1 small interfering RNA (RIPK1 siRNA), miR-141-3p mimics, miR-141-3p inhibitor, the relevant negative control (NC), pcDNA3.1-RIPK1 and pcDNA3.1 were purchased from Sangon (China), and the sequences were shown in Table 1. Lipofectamine 3000 (Invitrogen, USA) was used to conduct transfection in line with the manufacturer’s guidelines.

### Detection of cell viability

Cell Counting Kit-8 (CCK-8, Beyotime, China) was utilized to measure cell viability. In brief, 6000 cells were seeded into each well in a 96-well plate followed by indicated treatment for 24 h. 110 ml culture medium containing 10 ml reagent was added into each well. After 2 h, optical density (OD) 450 was recorded by Microplate Reader (Bio-Rad, USA).

### Propidium iodide (PI) staining

After indicated treatment, Caco-2 cells in the 6-well plate were washed and stained with PI solution (Invitrogen, USA) for 20 min in the dark. After being washed, the cells were stained with DAPI to mark the nuclei in the dark. The PI positive cells represented necrotic cells that carried red fluorescence.

### Luciferase reporter gene assay

TargetScanHuman 7.2 database was used to acquire the binding sites between miR-141-3p and wild type RIPK1. The procedures of luciferase assay were performed according to the previous study [19]. In our research, co-transfection of the indicated vectors (Sangon, China) was conducted by using Lipofectamine 3000 (Invitrogen, USA) in Caco-2 cells. Bio-Glo^TM^ Luciferase Assay System (Promega, USA) was utilized to evaluate the luciferase activity.

### Western blot

RIPA Lysis Buffer (Beyotime, China) containing PMSF (Beyotime, China) and PhosSTOP (Sigma-Aldrich, USA) was used to lyse the cells with indicated treatment. After quantification, the protein samples were separated by SDA-PAGE gel (Beyotime, China). Primary antibodies (Anti-RIPK1, BD Biosciences, USA; Anti-RIPK3, Abcam, USA; p-RIPK3, Abcam, USA; Anti-MLKL, Sigma-Aldrich, USA; Anti-p-MLKL, Sigma-Aldrich, USA; Anti-GAPDH, Beyotime, China) were used at 4 °C overnight. The protein bands were detected by ECL Kit (BOSTER, China).

### Immunoprecipitation (IP) assay

Pierce Co-Immunoprecipitation Kit (Thermo Fisher Scientific, USA) was used to detect interaction of RIPK1 and RIPK3 in accordance with the guidelines. Briefly, the cells with indicated treatment were harvested and lysed by IP Lysis Buffer. The primary antibody RIPK1 (BD Biosciences, USA) was added into the lysis solution containing the total protein, followed by using Sodium cyanoborohydride, Coupling Buffer, Elution Buffer and Loading Buffer, to obtain the experimental samples. RIPK1 was used to the control of IP, and the expression of RIPK1 and RIPK3 was detected by western blot analysis.

### Statistical analysis

The data in our research were represented as mean ± standard deviation (SD) and analyzed by GraphPad Prism 8 Software (GraphPad, USA). The unpaired *t*-test was used. *p* value less than 0.05 was regarded as statistical significance.
